# Crack-Tip Strain Field Mapping and the Toughness of Metallic Glasses

**DOI:** 10.1371/journal.pone.0083289

**Published:** 2013-12-27

**Authors:** Todd C. Hufnagel, Uday K. Vempati, Jonathan D. Almer

**Affiliations:** 1 Department of Materials Science and Engineering, Johns Hopkins University, Baltimore, Maryland, United States of America; 2 Advanced Photon Source, Argonne National Laboratory, Argonne, Illinois, United States of America; King's College London, United Kingdom

## Abstract

We have used high-energy x-ray scattering to map the strain fields around crack tips in fracture specimens of a bulk metallic glass under load at room temperature and below. From the measured strain fields we can calculate the components of the stress tensor as a function of position and determine the size and shape of the plastic process zone around the crack tip. Specimens tested at room temperature develop substantial plastic zones and achieve high stress intensities (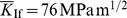) prior to fracture. Specimens tested at cryogenic temperatures fail at reduced but still substantial stress intensities (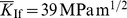) and show only limited evidence of crack-tip plasticity. We propose that the difference in behavior is associated with changes in the flow stress and elastic constants, which influence the number density of shear bands in the plastic zone and thus the strain required to initiate fracture on an individual band. A secondary effect is a change in the triaxial state of stress around the crack tip due to the temperature dependence of Poisson's ratio. It is likely that this ability to map elastic strains on the microscale will be useful in other contexts, although interpreting shifts in the position of the scattering peaks in amorphous materials in terms of elastic strains must be done with caution.

## Introduction

Fracture behavior is important for many prospective engineering applications of metallic glasses. Unlike conventional crystalline alloys for which stable flow in tension is possible due to strain hardening resulting from dislocation activity, metallic glasses strain soften during plastic deformation leading to localization of flow into shear bands. The result is apparently brittle mechanical behavior with almost no ductility in tension in most cases. Microscopically, however, there is often clear evidence for extensive plastic deformation of the material near the crack path. This allows some metallic glasses to achieve high values of fracture toughness despite the lack of macroscopic ductility [1], [2].

In any material there is a competition between flow (driven by shear stresses) and cleavage (driven by normal stresses), the outcome of which influences the stress intensity at the crack tip and determines the fracture toughness [3]. In the case of metallic glasses, crack-tip blunting may occur by either homogeneous or inhomogeneous plastic flow, both of which are strongly affected by the stress state around the crack tip. In addition, hydrostatic stresses near the crack tip may promote cavitation as an alternative ductile fracture mechanism.

Several groups have previously examined the plastic zone in metallic glasses using shear band patterns on the surface [4], [5]. Others have noted a strong correlation between the size of “vein” or “river” pattern features on the fracture surface (which are presumed to be related to the size of the plastic zone) and the fracture toughness over several orders of magnitude of each [6]. This observation suggests that the fracture toughness of metallic glasses scales only with the size of the plastic zone and is insensitive to details of the mechanism of fracture. Indeed, as the plastic zone size approaches the atomic scale the fracture energy approaches the Griffith limit for a brittle material [1].

The present work is motivated by a desire for a deeper understanding of the fracture behavior of metallic glasses, and in particular the influence of stress and strain fields on competing mechanisms of deformation and fracture near the crack tip. To this end, we have conducted fracture toughness tests on specimens of a Zr-based metallic glass using *in situ* high-energy synchrotron x-ray scattering to map out the strain field around the crack tip as a function of applied stress intensity at various temperatures well below the glass transition. This technique allows us to probe the entire volume of material, including the plane-strain region in the interior of the specimen around the crack tip. An analysis of the strain maps (and corresponding maps of stress) allows us to determine the size and shape of the plastic process zone around the crack tip. We observe that the extent of the plastic zone increases, as expected, with applied stress intensity. The plastic zone is reduced at cryogenic temperatures, an observation that is correlated with a reduction in fracture toughness.

Although plastic deformation of metallic glasses has been studied extensively, fracture has been less thoroughly investigated even though it is obviously of central importance for structural applications. Much of the early experimental work, limited as it was to studies of thin specimens not well suited to mechanical testing, focused on phenomenology and in particular on the development of the characteristic “river” patterns observed on fracture surfaces, and on the tendency for annealing to foster brittle behavior due to either structural relaxation or devitrification. A more fundamental understanding of fracture of metallic glasses developed from the work of Spaepen [7] and Argon and Salama [8] who described the development of river patterns as arising from an instability of a deforming thin fluid against fluctuations in density. Such a mechanism clearly invokes significant flow of the material around the advancing crack tip, but not all glasses show this behavior. Steif [9] used free volume theory to develop a constitutive law describing flow in the stress field around a crack tip, and showed that an increase in viscosity (as, for example, due to structural relaxation by annealing) limits the ability of the material to relax crack-tip stresses by flow, favoring brittle fracture. Wu and Spaepen [10] clearly demonstrated the existence of a ductile-to-brittle transition with decreasing temperature in an Fe-based metallic glass and were able to correlate the temperature at which the transition occurred with the degree of structural relaxation induced by annealing.

The development of bulk metallic glasses has enabled new studies with specimens appropriate for fracture mechanics experiments, and in particular proper plane-strain fracture toughness measurements. Gilbert and coworkers [11] measured a fracture toughness of 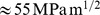 on fatigue-precracked specimens of 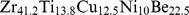 in a compact tension geometry but noted that fracture toughness predicted on the basis of the instability model of Argon and Salama [8] is only 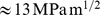. Flores and Dauskardt [4] demonstrated that the difference is due to the effect of branching at the crack tip; the stress intensity at the tips of the individual branches is consistent with the predictions of the instability model and is much lower than the far-field stress intensity factor.

On the basis of this work it is clear that the strain state around the crack tip is of central importance for understanding fracture of metallic glasses. To probe the strain state in a spatially-resolved way we employ high-energy x-ray scattering. Although there is a long history of using scattering techniques to measure elastic strains in crystalline materials [12], applications to amorphous materials were rare (see Ref. [13] for an interesting early example) until Poulsen and coworkers [14] demonstrated the ability to measure strains in a metallic glass using x-ray scattering from either shifts in the positions of the scattering maximum in reciprocal space or from shifts in peak positions in the radial distribution function (RDF) in real space. Generally speaking, the strains from scattering measurements are in reasonable agreement with the strains calculated on the basis of the known load and elastic constants known from other techniques (typically ultrasound), although in some cases there do appear to be discrepancies that may be related to fundamental differences in the physical basis of the two kinds of measurement [15]. One cautionary note is that the measurement of elastic strains from shifts in peak position in reciprocal space makes an implicit assumption that the deformation is homogeneous and does not involve a significant alteration of the atomic-scale structure of the material. This assumption can be violated in several ways, for example if there is significant plastic deformation [16], [17], and in such cases a simple peak shift may no longer be a reliable measure of elastic strain. Even for nominally elastic deformation the microscopic strains measured from scattering can show significant non-linearity as the macroscopic yield stress is approached [18]. This complicates the interpretation of our results, as will be discussed below.

An important feature of the high-energy x-ray technique is that it allows us to make a direct assessment of the strain state in a thick specimen, including the interior region ahead of the crack tip that is in a state of plane strain. This is in contrast to earlier work that examined the plastic zone using shear band patterns on the surface [4], [5] (which is necessarily in a state of plane stress) or which inferred the size of the plastic zone indirectly from the scale of features on the fracture surface itself [6].

## Results

### Stress intensity at fracture and fracture surface morphology

**Figure 1:**
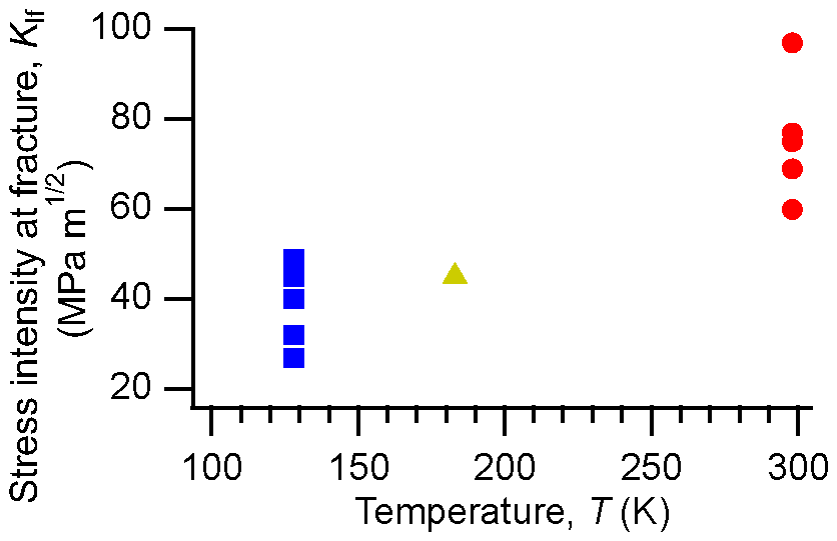
Loss of toughness with decreasing temperature in 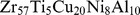.

The stress intensity at fracture from our tests on 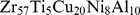 is shown in Fig. 1. Because we did not strictly follow standard techniques for plane-strain fracture toughness testing (as discussed in the Methods and Materials section below) we refer to the data in Fig. 1 as apparent fracture toughnesses or the stress intensities at fracture (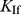) rather than true mode I plane strain critical stress intensities (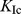). Figure 1 demonstrates a significant drop in apparent toughness with decreasing temperature, with the average stress intensity at fracture falling from 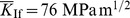 at room temperature to 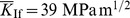 at the lowest temperature tested (128 K). We note that the room-temperature average is consistent with other measurements on fatigue pre-cracked specimens on glasses of similar composition [5], [19].

The degree of scatter in Fig. 1 is common in fracture toughness measurements on metallic glasses and is sometimes attributed to the presence of crystallites in the glass [5], [19]. In our work we can examine this question, because the synchrotron x-ray scattering is sensitive to the presence of small volume fractions of crystallites and specifically probes the local region around the crack tip. Although we did occasionally observe crystalline peaks in the data they were rare, with most specimens showing no evidence of crystals at all. Only on one specimen did we observe evidence of crystallization more than a few percent of the regions probed. Data from that specimen are not included in this report.

**Figure 2:**
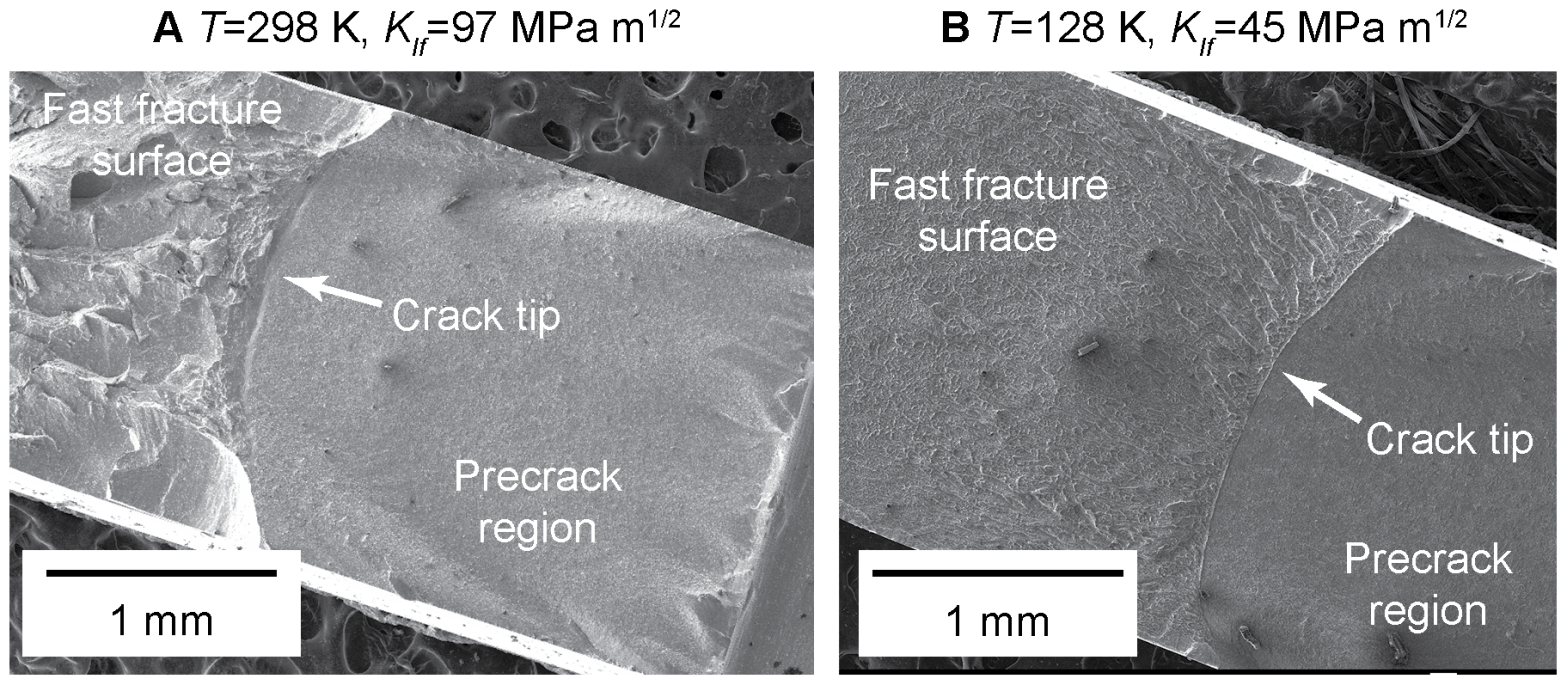
Fracture surfaces of SENB specimens of 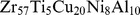 tested at (a) 298 K and (b) 128 K. The fatigue pre-cracking process produces a relatively flat fracture surface with a curved crack tip. When the specimen fails at the critical load, a fast-fracture surface morphology results that is much rougher for the specimen tested at room temperature than for the cryogenic specimen.

Fracture surfaces for specimens tested at room temperature and low temperature are shown in Fig. 2. The fatigue precracking process produces a relatively flat fracture surface, with a curved crack front resulting from the difference in stress state through the thickness of the specimen (plane stress at the surfaces and plane strain in the interior). The overload fracture surface is much rougher for specimens fractured at room temperature than for the specimens fractured at cryogenic temperature, implying a greater degree of plastic deformation. The roughness of the fracture surface apparently correlates with the fracture toughness, as has been previously reported by others [20].

### Crack tip strain and stress fields

**Figure 3:**
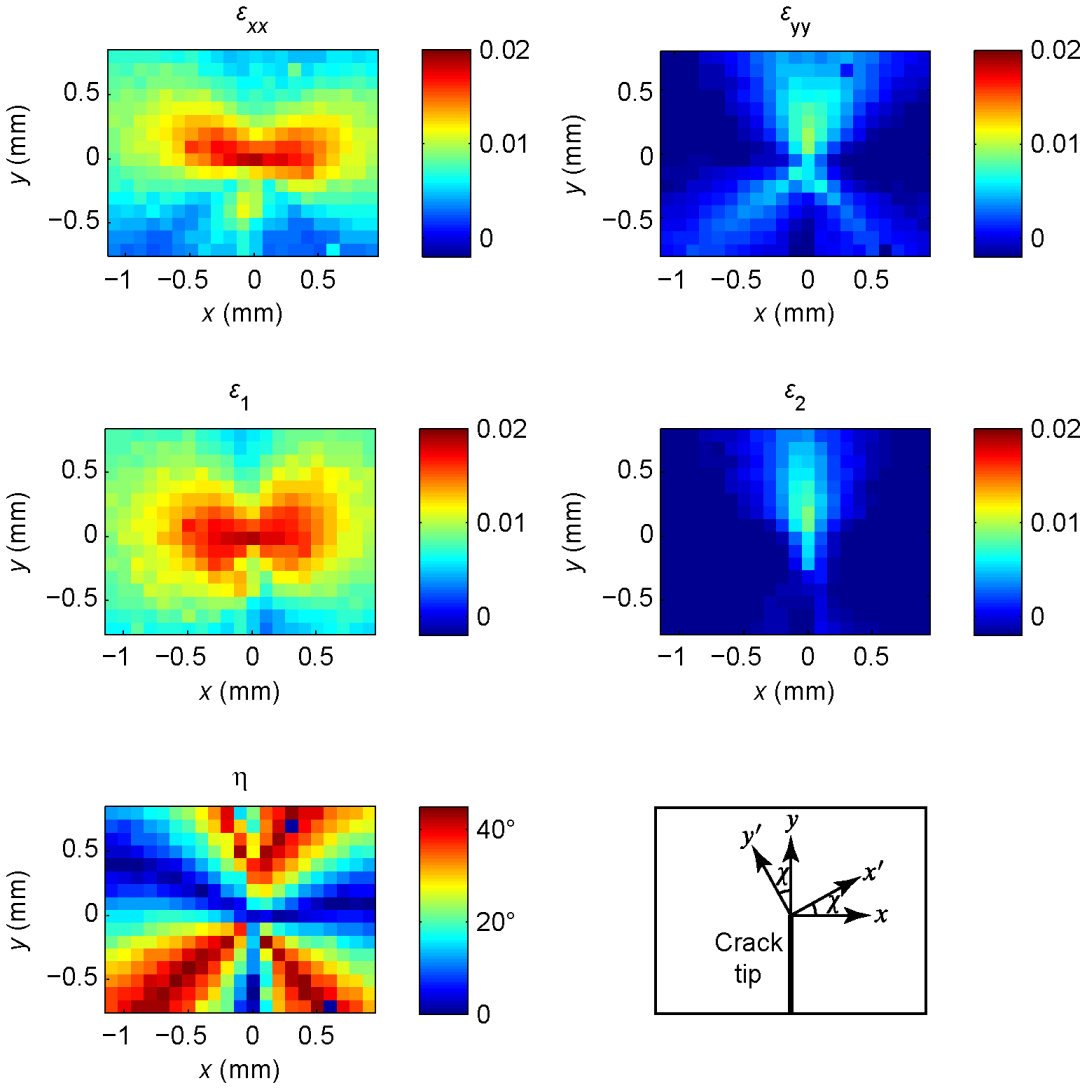
Strain maps around the crack tip of an amorphous 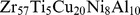 specimen loaded in the SENB geometry loaded to a stress intensity 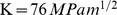 at room temperature. Top row: Normal strains in the laboratory coordinate system, 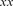 and 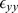. Middle: Principal strains 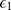 and 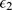, the orientation of which defines the 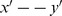 coordinate system. Bottom: Orientation of principal axes with respect to the lab coordinate system, 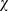. See also Fig. 9.

Using the analysis outlined in the Methods and Materials section below we can deduce the components of strain perpendicular to the x-ray beam as functions of position around the crack tip. In particular, we can determine the normal strains in the laboratory coordinate system (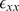 and 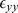) as well as the two principal strains 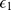 and 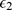 in the plane perpendicular to the x-ray beam. Figure 3 shows these strains mapped out around a crack tip in a specimen under a high load 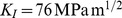 at room temperature. Notice that the coordinate system used here is drawn from the x-ray literature, which typically makes the 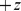 the direction of propagation of the incident beam. Given this and the geometry described in the Methods and Materials section below, the crack-opening normal strain is 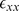. In the fracture literature it is usual to make the 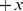 direction the direction of crack propagation, meaning that the crack-opening strain is 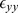. Using these strain measurements (under the assumption of plane strain as discussed in the Methods and Materials section) with the elastic constants from Table 1 we can now calculate all of the components of the stress tensor along with any derived quantities. For example, the von Mises effective shear stress is defined as 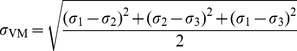
(1)where 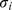 are the principal stresses, and the mean or hydrostatic stress is 
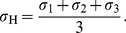
(2)


**Table 1:** Elastic constants.

Temperature (K)	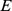 (GPa)	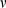
298	86.8	0.368
183	88.8	0.366
128	89.8	0.365

Young's modulus 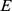 and Poisson ratio 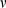 used for the stress calculations. Room-temperature data are from Ref. [39] and the temperature dependence is estimated from data in Ref. [40].

**Figure 4:**
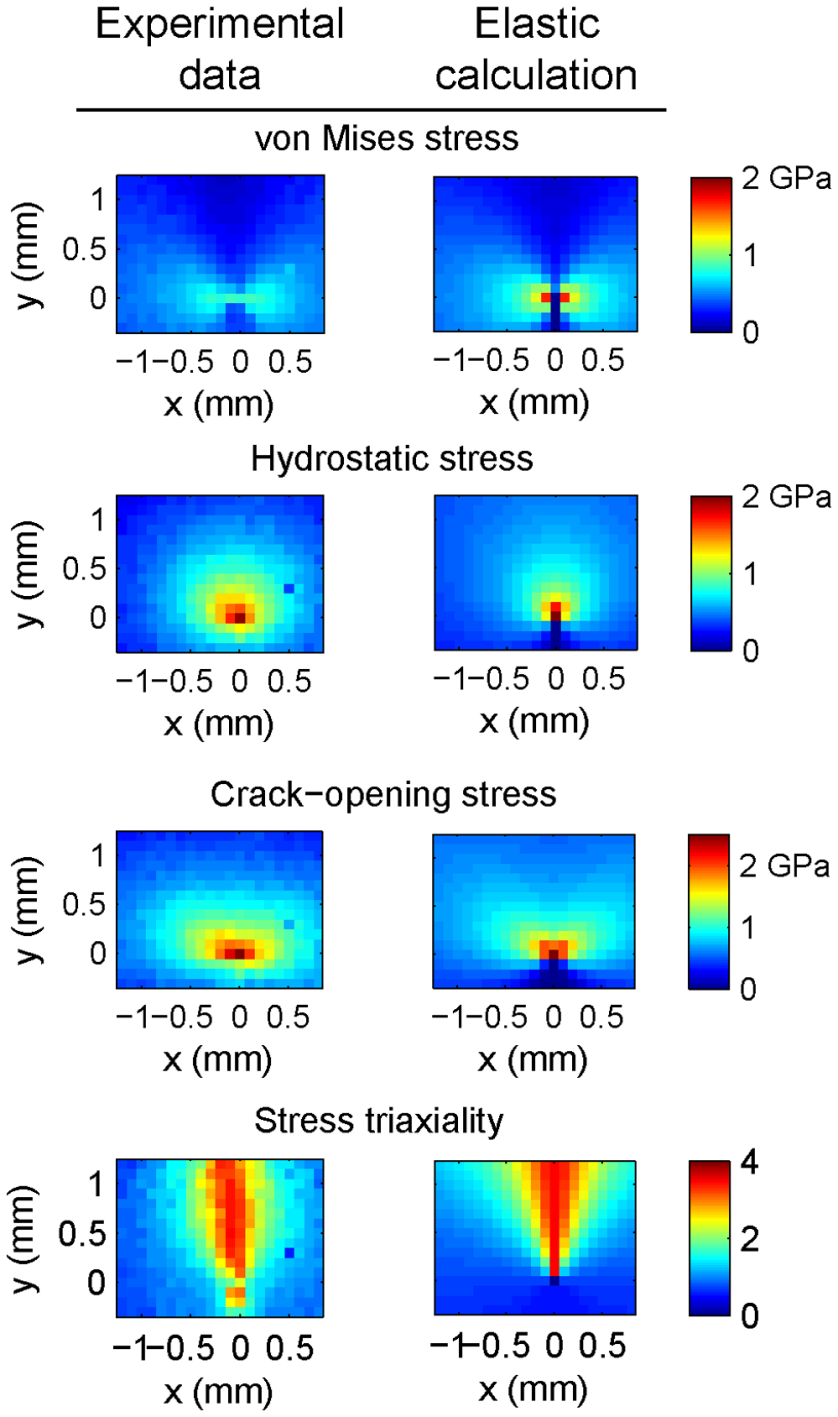
Comparison of crack-tip stress fields from experimental data (left-hand side of each pair) with a fully elastic, plane-strain calculation for amorphous 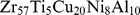 loaded to 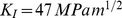 at room temperature.

Figure 4 shows each of these along with the crack-opening normal stress 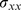 and the stress triaxiality factor 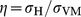 for a room temperature specimen at a stress intensity of 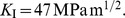


As a check on our results, we compare the stresses determined from the x-ray data with a calculation based on a simple model for the stresses around a crack tip in mode I loading in an infinite, fully elastic body [21] (also shown in Fig. 4). Even at the lowest stress intensities considered in this work we expect there to be some plastic deformation around the crack tip, so it would be unreasonable to expect such a simple model to be quantitatively correct within or near the plastic zone (which, as we show below, has characteristic dimensions of a few hundred microns under these conditions). At the other extreme this simple model does not work when the distance from the crack tip approaches the dimensions of the specimen.

For all four quantities compared (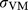, 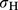, 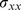, and 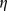) the data sets and the calculations show similar features. (Below we show that the stresses extracted from the x-ray data show the 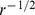 behavior expected of an elastic material well away from the crack tip.) For example, the “butterfly” shape of the lobes in the von Mises effective stress is characteristic of plane strain conditions. As expected the stress concentration near the crack for the experimental data is less intense than that of the elastic solution (for which the stresses become infinite as 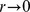). Because the stress concentration at the crack tip is smaller, the stress field extends out to larger distances for the experimental data than for the elastic calculation.

It is particularly interesting to note that directly ahead of the crack tip (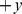 in Fig. 4) there is excellent quantitative agreement between the measured triaxiality (which has a maximum of 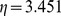) and that calculated from the model (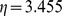). For both the x-ray stress determination and the elastic model the particular value of the triaxiality depends strongly on the value of Poisson's ratio chosen, but the fact that the model and data do agree is strong evidence that the x-ray technique measures the strain in a fundamentally correct way, at least outside of the plastic zone.

### Plastic zone around crack tips

**Figure 5:**
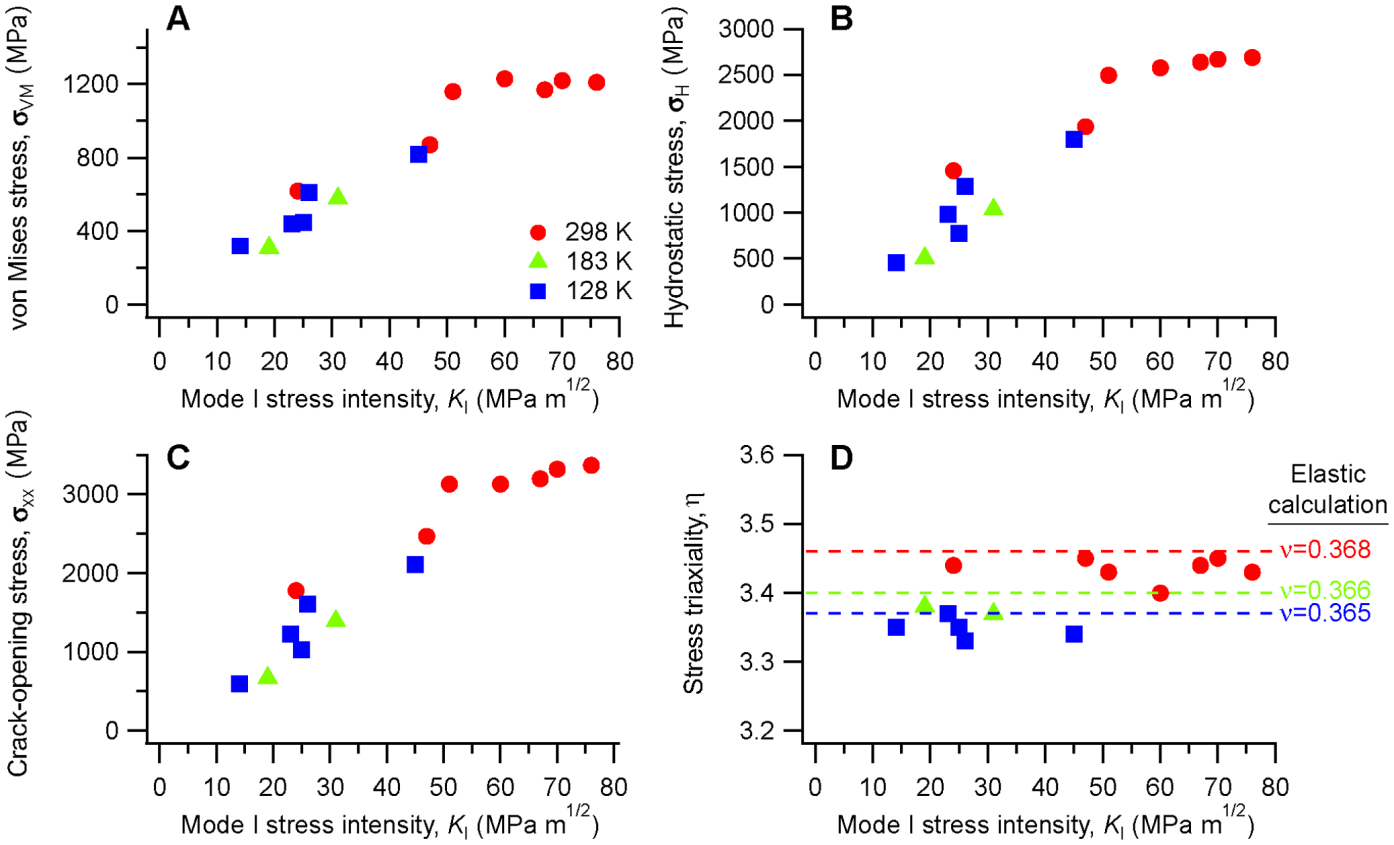
Trends in measured (a) maximum effective (von Mises) stress 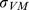, (b) mean (hydrostatic) stress 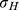, (c) crack-opening normal stress 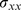, and (d) stress triaxiality 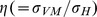 as functions of stress intensity 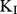 for multiple specimens at three temperatures. The dashed lines in (d) are the triaxialities calculated from the stress fields ahead of a crack tip in an elastic material under plane strain, using the values of 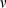 from Table 1 for the three temperatures.

With the ability to measure strains and stresses in a spatially-resolved way, we can examine trends that develop as a function of load, temperature, or both, using data collected from multiple specimens. We have found it convenient to look at the maximum value of the von Mises stress, hydrostatic stress, and stress triaxiality from each map (*i.e.* for a given stress intensity on a given specimen), and then compile these into plots showing the trends with stress intensity, as shown in Fig. 5. At low loads the behavior is fully elastic, with the various components of stress increasing linearly with stress intensity. At stress intensities above about 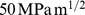 the stresses no longer increase, instead maintaining approximately constant values with increasing stress intensity. Only the specimens tested at room temperature show this behavior; all of the specimens tested at lower temperatures fractured at 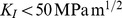.

This behavior is broadly consistent with that expected for metallic glasses, which demonstrate macroscopic mechanical behavior (at temperatures well below the glass transition) that is nominally elastic-perfectly plastic until fracture, with no strain hardening. We would expect a linear increase in the stress components with increasing stress intensity, except for the region immediately around the crack tip where plastic deformation can occur. Only when the size of the plastic zone exceeds the spatial resolution of the measurement would we expect to see the saturation in stress values apparent for 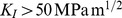.

The plateau in von Mises stress in Fig. 5 occurs at about 1200 MPa, lower than the expected room-temperature yield stress of 1750 MPa based on the uniaxial yield stress (Ref. [22]) under the assumption that the glass follows the von Mises yield criterion. A more appropriate yield criterion might incorporate some dependence on either the hydrostatic stress or the normal stresses [1] so the fact that we observe a reduced effective shear stress is not surprising. Furthermore, Das and coworkers observed a non-linear relationship between stress and strain (measured via scattering) in metallic glasses approaching the yield stress [18]. Although we have previously verified a linear relationship between x-ray strain and stress in this alloy up to about 60% of the yield stress [23], a similar effect here would affect the observed value of 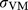 at higher stresses.

**Figure 6:**
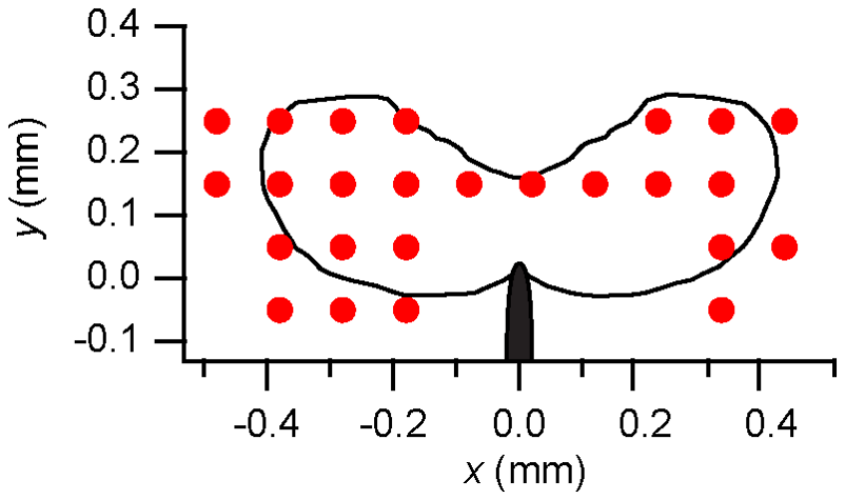
Points for which the von Mises stress is at the plateau value (Fig. 5) for a specimen loaded to 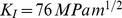 at 298 K. Also shown is the shape of the plastic zone from a finite element model for 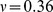 from Ref. [24].

The observation of a plateau in the stress components in Fig. 5 is evidence for the development of a substantial plastic zone around the crack tip and we use the extent of the region over which the von Mises stress reaches its plateau value as an estimate of the size of the plastic zone. Tandaiya and coworkers [24] have calculated the crack-tip strain fields for mode I fracture in a metallic glass, using a constitutive model for metallic glasses developed by Anand and Su [25]. Figure 6 compares their calculated plastic zone with the region determined from the x-ray scattering measurements to have achieved the plateau value of von Mises effective stress from one of the room-temperature specimens. (Because the location of the crack tip is not precisely known for the x-ray data, they have been shifted by 0.1 mm vertically to bring them approximately into coincidence with the calculation.) The apparent shape of the plastic zone from the scattering measurements is in rough agreement with the calculations, although it is somewhat larger in spatial extent.

**Figure 7:**
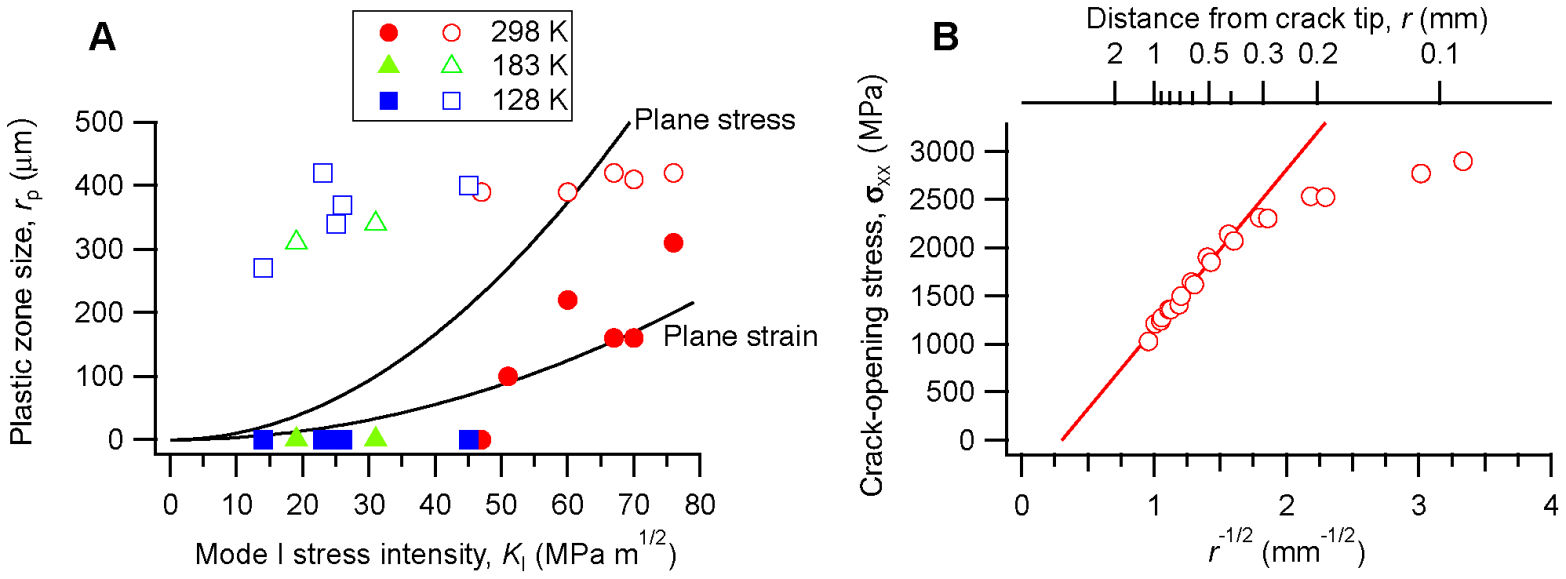
Plastic zones around crack tips. (a) The size of the plastic zone 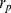 as determined in two ways: Solid symbols are from the radius of gyration 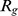 of the region for which the von Mises effective stress has achieved its plateau value (Eqn. 3), while open symbols are from 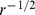 fits similar to those in part (b). The uncertainty in the plastic zone size is approximately 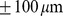 but for clarity error bars are not shown. For comparison, analytical estimates of 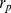 are shown for plane stress and plane strain conditions (Eqn. 4) using a room-temperature yield stress of 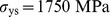. (b) Crack-opening normal stress 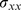 ahead of the crack tip for a specimen loaded to 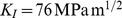 at 298 K. The straight line is a fit to the data for 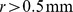.

A quantitative estimate of the plastic zone size can be made by calculating the radius of gyration (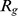) of the region of material around the crack tip for which the von Mises stress has reached its plateau value of 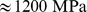 (Fig. 6). 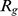 is calculated from 
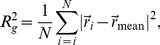
(3)where 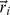 is a vector from the origin to the 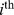 pixel for which the von Mises stress has reached the plateau value, and 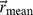 is the average position of all 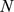 such pixels. This is plotted in Fig. 7(a) along with an analytical estimate of the extent of the plastic zone directly ahead of the crack tip, 
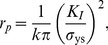
(4)where 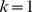 for plane stress conditions and 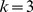 for plane strain [26]. The agreement is reasonably good for 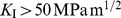, allowing for the uncertainty in the data and the fact that the experimental measurement averages a range of stress states through the thickness of the material.

Another way of determining the approximate size of the plastic zone makes use of the fact that all of the components of stress are proportional to 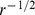 for the fully elastic solution [27]. If there is plasticity around the crack tip the stresses deviate from the 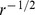 behavior close to the crack tip, but at distances sufficiently far from the crack tip the stresses still go as 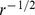. The distance from the crack tip at which the deviation from 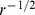 behavior occurs can be taken as a measure of the size of the plastic zone [27]. An example of this is shown in Fig. 7(b) where we see that 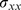 is indeed linear with 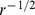 at large 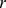 but that the proportionality breaks down at 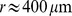. This distance is also shown in Fig. 7(a), but for reasons discussed below it should be interpreted with caution and is probably best viewed as an upper bound on the size of the plastic zone.

## Discussion

An important point to consider in evaluating our results is whether a peak shift in the scattered intensity 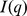 can in fact be used as a reliable measure of elastic strain. The available evidence suggests that it can, at least under loading conditions where there is little or no plasticity [14], [23], [28]. There is cause for concern, however, in situations where there is large-scale plasticity, which is associated with atomic rearrangements that can change the peak positions without an associated elastic strain [16], [17]. Furthermore, Das and coworkers [18] observed the stress-strain relationship under uniaxial compression [where strain was measured from shifts in 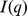] became significantly nonlinear even for nominally elastic loading when the stresses approached the yield stress of the glass. It is possible that similar effects are at play here, for example in our observation that the plateau value of the von Mises stress is lower than expected.

Bearing these caveats in mind, our results indicate that x-ray scattering can be used to map out the strains and thus stresses around crack tips in metallic glasses (and possibly in other amorphous materials as well). In particular, we observe an 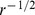 dependence of the stresses away from the plastic zone, in agreement with classic models of crack-tip stress fields. Whether or not the values of stress are quantitatively accurate depends in part on our knowledge of the appropriate elastic constants to use in calculating the stresses from the measured strains. Although the x-ray elastic constants for this alloy appear to be in reasonable agreement with elastic constants measured in other ways [23], this is not necessarily the case for other alloys [15]. Furthermore, for our low-temperature measurements we have extrapolated from the room-temperature data on the basis of the expected behavior of the elastic constants, but there is no guarantee that this extrapolation is correct.

Our results show that the fracture toughness of amorphous 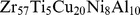 is reduced at low temperatures (Fig. 1). Based on observations of fracture surface features, Xi and coworkers proposed that the toughness of metallic glasses scales with the size of the plastic zone [6]. Our results provide a more direct assessment of the plastic zone size. As Fig. 7(a) shows we do not observe a large plastic zone in the low-temperature specimens, but we note that a small plastic zone (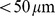) would be below the spatial resolution of our measurement and would probably not be detected in these experiments. Furthermore, the apparent fracture toughness at low temperature is still relatively high (at least compared to the Griffith limit for truly brittle fracture) again suggesting some plasticity.

Our observation that the crack-tip stresses do deviate from 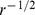 behavior close to the crack tip, even for the low-temperature specimens, should not be used as direct evidence for the existence of a plastic zone because the relationship between strain (derived from x-ray peak shifts) and stress can become nonlinear at high stresses [18]. This is why we view the distance at which deviation from 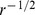 behavior is observed as an upper bound on the plastic zone size. We observe that it is always larger than the plastic zone size estimated from the extent of the plateau in von Mises stress [Fig. 7(b)] and that it is only weakly dependent on the stress intensity. Even the specimens at low 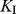 for which the von Mises stresses do not reach the plateau show deviations from 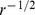 behavior at significant distances (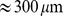) from the crack tip. We suspect that this is due in part to complicating effects such as the curved crack tip and non-flat crack surface.

Our data clearly show that a reduction in fracture toughness at low temperatures is associated with a reduction in the size of the plastic zone. Part of the explanation for this is that the flow stress of Zr-based metallic glasses increases with decreasing temperature, by amounts on the order of 15–20% [29]. On the basis of Eqn. 4 we would therefore expect the size of the plastic zone would be reduced by approximately 40% for the same stress intensity, compared to room temperature. Given the stress intensities at which the low-temperature specimens failed (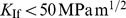) the plastic zone size would be less than 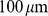 in size [Fig. 7(a)] and probably not observable in these experiments.

Tandaiya and coworkers recently proposed that fracture in ductile metallic glasses is not stress-controlled but rather is a strain-controlled process [30]. They reported that initiation of fracture in notched specimens requires that a critical shear displacement be attained on an individual shear band. Although the conditions in our specimens (which were fatigue-precracked) are somewhat different, it is worth considering whether this mechanism is consistent with our results.

If attainment of a critical strain on an individual band is required for fracture, the number density of shear bands around the crack tip becomes an important consideration because a larger number of bands implies a smaller strain on each individual band, at least on average, for a given overall level of strain. Ravichandran and Molinari [31] showed that for metallic glasses in bending the number density of shear bands 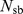 is related to both the elastic constants and the flow stress 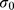 of the glass: 
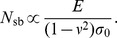



Although the bending geometry is not directly applicable to the mode I opening around the crack tip in our experiments, we assume that a similar scaling relationship holds. With increasing temperature the flow stress of metallic glasses decreases significantly [29]; 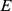 also decreases and 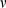 increases, but only slightly (Table 1). Taken together these suggest that for the same imposed strain the shear band density increases with increasing temperature. With more shear bands on which to distribute the imposed strain at the crack tip, the strain on the individual bands would, on average, be smaller. This in turn suggests that reaching the critical shear displacement at which a shear band becomes a crack requires a larger imposed strain or, in other words, a larger plastic zone. This is consistent with our observation that the plastic zone is significantly larger in the room-temperature specimens. Conversely, the low-temperature specimens have fewer shear bands in the plastic zone, concentrating the shear on a few bands and allowing the critical strain to cause fracture to be reached in a smaller plastic zone.

Several groups have reported that tough metallic glasses tend to have high values of 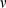 or, equivalently, low ratios of shear modulus to bulk modulus [32], [33]. This observation has been rationalized in terms of a competition between plastic flow (which is related to the shear modulus) and the dilation necessary for fracture (which is related to the bulk modulus) [1]. It is not clear to us that small changes in Poisson's ratio due to changing temperature significantly alter this balance.

However, we also note that 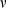 can have an indirect effect on the competition between flow and fracture though its influence on the stress state ahead of the crack tip. Increasing Poisson's ratio has the effect of increasing the stress triaxiality ahead of the crack tip [Fig. 5(d)]. The significance of increased triaxiality is twofold: It promotes cavitation (a ductile fracture mode) ahead of the crack tip and it facilitates diffusive annihilation of free volume generated by plastic deformation of the metallic glass. Although the latter effect could be viewed as opposing plastic flow, it might allow steady-state plastic flow around the crack tip without accumulation of a critical level of free volume that would lead to unstable fracture.

More generally, we believe that the x-ray strain mapping technique demonstrated here can be broadly applied to amorphous materials in many contexts. A key concern for such studies will be the conditions under which shifts in the scattering peak position can be correctly interpreted in terms of elastic strain, and the precise nature of the relationship between the strains measured from such peak shifts and the stress state of the material. Although the elastic constants inferred from x-ray scattering strain measurements are in rough agreement with those measured via ultrasound, there may be systematic differences in some cases [15] and at high stresses careful attention must be paid to whether the relationship between stress and x-ray strain is linear [18]. Complicating matters further is the observation that the elastic constants of metallic glasses depend on the hydrostatic stress [34].

## Materials and Methods

### Experimental

**Figure 8:**
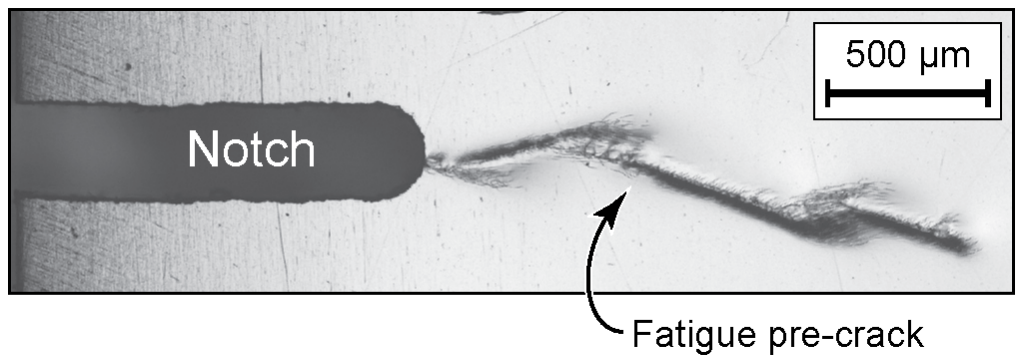
Optical micrograph showing the surface of a specimen after fatigue precracking.

The specimens for this study were single edge-notch bend (SENB) samples of a Zr-based metallic glass. We prepared amorphous specimens of nominal composition 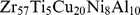
by arc melting the pure elements and then suction-casting the alloy melt into plates of nominal dimensions 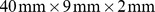. Additional details of the melting and casting process can be found in Reference [35]. After casting, each plate was mechanically polished to a 1200 grit finish using SiC grinding paper, and a notch approximately 0.3 mm wide by 2 mm deep was cut in the center of the long side of each specimen with a low-speed diamond saw. Finally, the specimens were subjected to fatigue loading at room temperature at 2 Hz under a stress ratio of 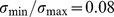 with a maximum stress intensity of 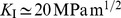 to produce fatigue precracks approximately 2 mm long. As shown in Fig. 8 the fatigue cracks are accompanied by significant shear band activity over length scales of about 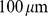. Although the crack does not appear flat in this view, this is a surface effect. The pre-crack fracture surface in the interior of the specimen [Fig. 2(a)] is flatter than this view would suggest.

**Figure 9:**
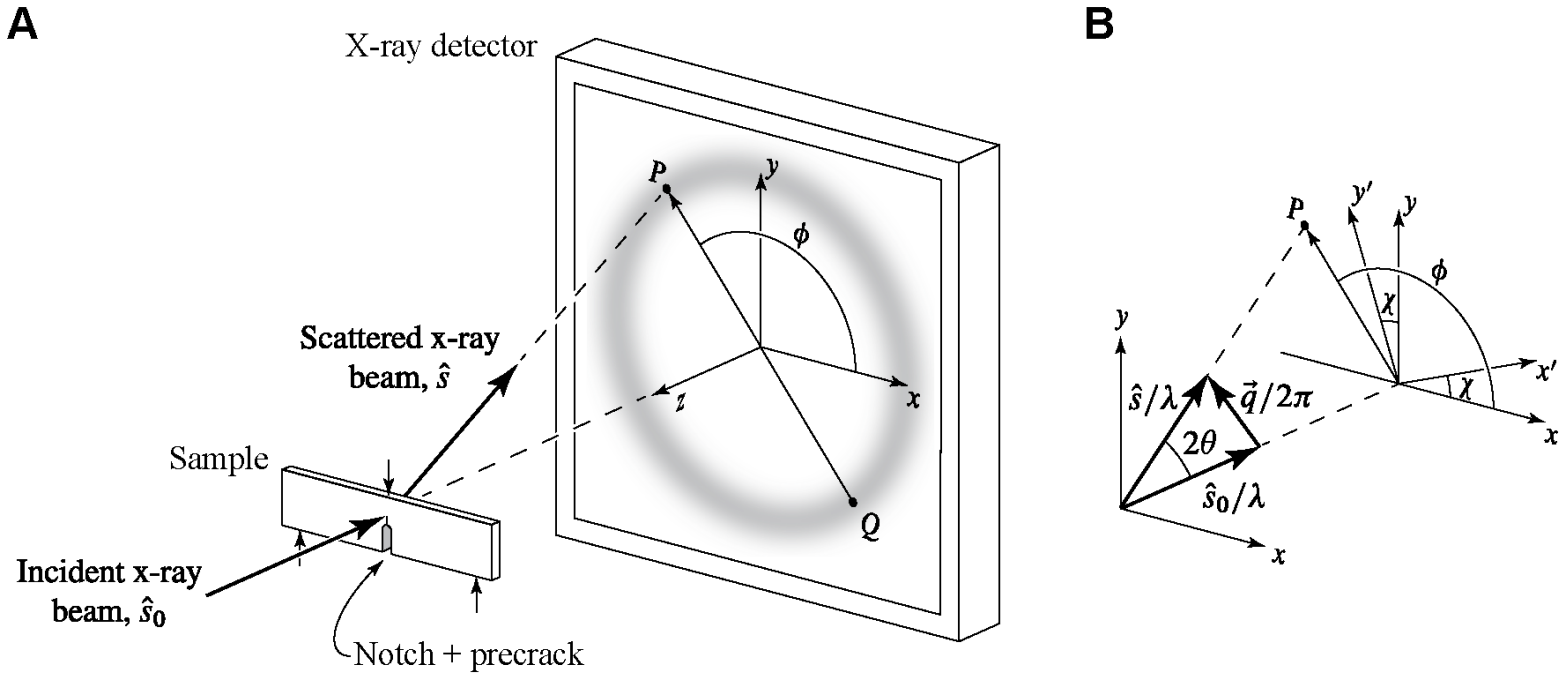
Experimental geometry and coordinate systems for the *in situ* studies of fracture. (a) The single-edge notched bend (SENB) specimen is loaded in three-point bending using a load frame that can be translated in both horizontal (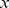) and vertical (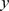) directions. A high-energy x-ray beam with a cross section of 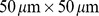 is used to produce transmission scattering patterns as a function of position relative to the crack tip. From changes in the diameter of the scattering ring 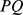 the strain as a function of orientation 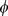 relative to the laboratory (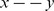) coordinate system can be determined. (b) Structural information comes from a direction determined by the scattering vector 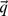. Because 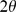 is small (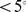), 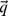 lies nearly in the 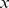
–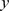 plane, allowing determination of the normal strains 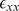 and 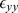 as well as the shear strain 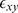. The principal axes of strain 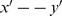 are oriented at an initially unknown angle 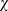 relative to the laboratory (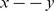) coordinate system. No information is available about the strain components in the 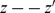 direction parallel to the incident beam.

The x-ray scattering experiments were performed at beamline 1-ID of the Advanced Photon Source (APS), with the SENB specimens loaded in three-point bending (outer span 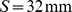) using a hydraulic load frame (Fig. 9). The load frame was mounted on a table capable of translation perpendicular to the x-ray beam in both horizontal and vertical directions, allowing us to position the incident x-ray beam relative to the crack tip in the specimen. The x-ray scattering experiments were performed with 86 keV x-ray s, with scattering in transmission through the specimen recorded by a GE amorphous silicon position-sensitive x-ray detector positioned approximately 750 mm downstream of the specimen. The x-ray beam size was 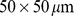 which, together with a typical translation step size of 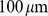, determines the spatial resolution of the strain mapping. To enable measurements at sub-ambient temperatures the experiments were carried out in an environmental chamber in which the specimens could be cooled by introducing cold nitrogen vapor. The temperature was measured by means of a thermocouple located near the specimen, and the chamber temperature was allowed to equilibrate prior to the scattering experiments.

Although our procedures were in general accord with ASTM standard E399 for fracture toughness testing of metals [36], measuring fracture toughness *per se* was not our primary aim and we did not adhere strictly to this test method. For example, E399 calls for loading the specimen monotonically until fracture but we loaded our specimens incrementally with pauses to record the x-ray scattering data. Also, because we did not record load-displacement data during loading we could not apply the secant technique from Ref. [36]. Instead, the apparent fracture toughnesses are based on the manually-noted load at fracture, following the calculations in E399, and therefore probably overestimate the fracture toughness by 5–10%.

A valid plane-strain fracture toughness measurement (according to ASTM E399) requires that the specimen be sufficiently thick that plane-strain conditions predominate. The specified minimum thickness is 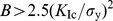, where 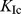 is the mode I plane-strain fracture toughness and 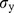 is the yield stress [36]. Given a room-temperature yield stress of 1750 MPa (Ref. [22]) and a thickness of 2 mm plane-strain conditions are satisfied for fracture toughness 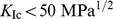. As described in the Results section above the apparent fracture toughness of some of specimens tested at room temperature exceeds this value, so plane-strain conditions are not fully satisfied for these specimens. At lower temperatures where the fracture toughness is reduced all of our specimens satisfied plane-strain conditions.

### Data analysis

The real-space structural information obtained in a scattering measurement is from a direction parallel to the scattering vector 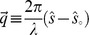, where 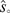 and 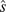 are unit vectors defining the direction of propagation of the incident and scattered x-ray s, respectively, and 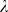 is the x-ray wavelength (Fig. 9). Because the x-ray detector in our experiment records scattering from all azimuthal angles 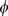 we can obtain structural information about any orientation perpendicular to the incident x-ray beam. The scattering vector 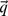 is actually off of perpendicular by 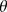, but because the wavelength of the high-energy x-ray s used in these experiments is small (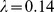
Å for 86 keV x-ray s) 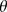 is also small (about 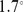) and we ignore this difference.

Elastic strain in amorphous alloys can be determined directly from shifts in the position of the first maximum in the scattering pattern [14], [23], [28]. To do so, we used the software package fit2d to azimuthally integrate the two-dimensional scattering data from the area detector into 72 one-dimensional scattering patterns, each corresponding to a unique 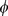 about the incident beam [37]. These patterns are the scattering intensity 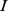 as a function of distance 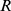 on the detector from the center of the scattering ring (which is the position of the direct beam). From basic geometry one can convert these data from 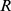 to the magnitude of the scattering vector 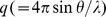, but because in calculating strain we are interested in peak shifts and not absolute positions this conversion is unnecessary.

We determined the positions of the first scattering maximum 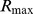 for each pattern by fitting the top of the peak to a Gaussian function. To avoid systematic errors associated with uncertainty in the position of the direct beam (which defines 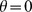), we use pairs of diametrically-opposed points to determine the diameter of the scattering ring 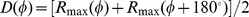 [distance 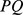 on Fig. 9(a)]. For purposes of strain measurement there is no distinction to be made between the 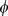 and 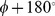 directions.

We can determine the normal component of elastic strain 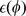 from the diameter measured with the specimen under load 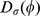 and the diameter measured for an unloaded specimen, 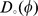: 
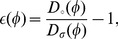
(5)as discussed in Ref. [23]. One potentially complicating factor is determining the appropriate value of the scattering ring diameter for an unstrained specimen, 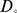 (which is equivalent to the problem of determining the unstrained lattice parameter in strain measurements of crystalline materials). We used values of 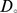 measured from each samples to be tested a position well away 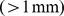 from the precrack tip, at the temperature of interest. Under these conditions the scattering rings were observed to be circular, with no apparent dependence of 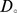 on the azimuthal angle 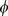. This suggests that any residual stresses due to casting, which could influence the results, are small.

Although there is considerable uncertainty in the individual 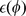 values in Eqn. 5, by using values for all 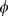 simultaneously one can reliably determine the strain components perpendicular to the incident beam. For example, from a plot of 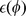 *v.* 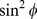 the normal strain components in the horizontal (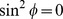) and vertical (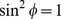) directions can be obtained; this approach is convenient for simple situations such as uniaxial loading [28].

For more complex strain states we determine the principal strains as well as the rotation of the principal axes relative to the laboratory coordinate system [Fig. 9(b)] from the measured 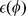 data. To do so, we first write an expression for the normal strains in the laboratory (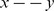) coordinate system in terms of the strains in the principal axes (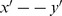) coordinate system and the angle of rotation 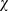 (about 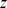) between the two sets of axes. Using the transformation rule for a second-rank tensor this expression is 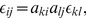
(6)where 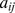 are the direction cosines between the 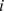 and 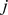 directions and summation over repeated indices is implied [38]. The components of strain of interest in the laboratory system are those corresponding to the 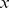 and 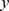 directions, perpendicular to the 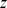 direction and therefore experimentally accessible to our x-ray scattering experiment [Fig. 9(b)]. Furthermore, we know that in the coordinate system corresponding to the principal strains there are no shear strains, so 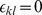 for 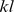. Therefore, the expressions for the nine components of strain (only six of which are independent) implicit in Eqn. 6, each with nine terms, are reduced to just three expressions of two terms each for the strains in the laboratory coordinate system: 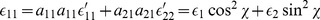
(7)

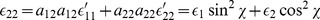
(8)

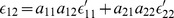


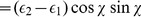
(9)where 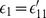 and 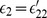 are the principal strains.

With these expressions we can now write a new expression for the experimentally-observed component of normal strain along the 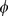 azimuth relative to the 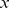 direction, again using the transformation rule. Using a double prime to indicate the coordinate system specified by a rotation of 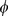 about the incident beam, this is 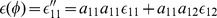


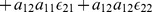


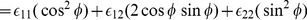
(10)where the 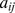 in the first line now refer to direction cosines between the laboratory coordinate system and the coordinate system rotated by 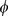, and we note that 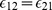. Using Eqns. 7–10 after some simplification we obtain 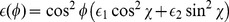
(11)

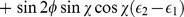


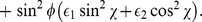



To find the principal strains and 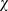 we fit Eqn. 11 to the measured 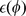 data with 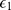, 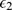, and 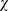 as the fitting parameters.

Due to limitations in the geometry of the experiment we cannot access any information about components of strain parallel to the x-ray beam (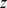 in Fig. 9). Calculation of the components of stress therefore requires an assumption about the conditions in this direction. The simplest choices are plane strain (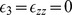) or plane stress (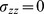). In reality, the truth lies somewhere in between. Even in a thin specimen the strain state near a crack tip varies rapidly with position, and our transmission x-ray scattering experiment inherently averages over regions near the surface (which is in plane stress) and plane strain (in the interior). Because the scattering angles are small, each volume element sampled by the x-ray beam in transiting the specimen is equally weighted. Examination of the fracture surfaces [Fig. 2(a)] and in particular the curved precrack front and the existence of shear lips (in the room-temperature specimen) suggests that both regions are substantial and thus make significant contributions to the x-ray scattering measurements. In this paper we assume plane strain to allow us to calculate all of the components of the stress tensor. Although this assumption influences the values we report, the qualitative trends do not change if a different assumption is made.

Once we have the principal strains (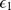, 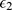, and 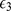) we can calculate the components of stress. Written in matrix form these are 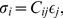
(12)where 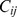 are the components of the stiffness matrix and summation over the repeated index 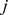 on the right-hand side is implied [38]. Doing so requires knowledge of two independent elastic constants for this (presumed) isotropic material in order that the 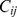 values may be determined. We are unaware of any reliable published elastic constants derived from ultrasound measurements for 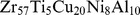 but values for the closely related amorphous alloy 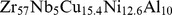 are available [39]. The elastic constants at different temperatures were estimated from results for for similar glasses reported in Ref. [40], assuming a linear dependence for both 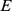 and 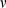 on temperature. The specific values used in the stress calculations are reported in Table 1. The choice of Young's modulus (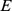) acts as a scaling factor on the stresses and has only a minor influence on the results. The effect of Poisson's ratio (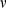) is more important because (under our assumption of plane strain) it strongly affects the stresses in the out-of-plane direction. In particular, a larger value of 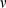 results in a larger stress triaxiality. This is illustrated by the dashed lines in Fig. 5(d) which show the triaxiality calculated from the stress field ahead of a crack tip in a fully elastic material for several values of 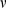.

## Conclusions

High-energy x-ray scattering can be used to examine the strain state locally around crack tips in metallic glasses, and presumably in other amorphous materials as well. Care must be taken in interpreting the data due to the complicating effects of plastic deformation and a nonlinear relationship between peak shifts and stress. In amorphous 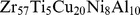 we observe the development of a substantial plastic zone for specimens tested at room temperature, which achieve apparent fracture toughnesses of 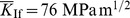, while specimens tested at 128 K do not develop such a large plastic zone and fail at lower stress intensities (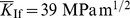). We propose that the difference is due to an increase in flow stress at low temperatures and possibly to a change in the stress state ahead of the crack tip, which becomes less triaxial at lower temperatures due to a decrease in Poisson's ratio. This strain-mapping technique can be applied to other amorphous materials, so long as care is taken in the interpretation of the scattering peak shifts.
